# Structural Design and Analysis of Large-Diameter D30 Conical Polycrystal Diamond Compact (PDC) Teeth under Engineering Rotary Mining Conditions

**DOI:** 10.3390/ma17020477

**Published:** 2024-01-19

**Authors:** Zhiling Xiao, Yuhao Zhang, Songhao Hu, Fan Zhang, Junjie Jiang, Hao Wang, Jiantao Li

**Affiliations:** 1 College of Mechanical and Electrical Engineering, Zhengzhou University of Light Industry, Zhengzhou 450002, China; vous200408@163.com (Y.Z.); violet5257@163.com (J.J.); 18247695198@163.com (H.W.); lijt7287@163.com (J.L.); 2 Industry & Innovation Research Center (IIRC), Zhengzhou University of Light Industry, Zhengzhou 450000, China; 3 Henan Huanghe Whirlwind Co., Ltd., Changge 461500, China; husongaho2008@126.com (S.H.); zzffan@163.com (F.Z.)

**Keywords:** conical PDC teeth, impact resistance, large diameter, simulation, crack extension

## Abstract

In the realm of engineering rotary excavation, the rigid and brittle nature of the Polycrystal Diamond Compact (PDC) layer poses challenges to the impact resistance of conical teeth. This hinders their widespread adoption and utilization. In this paper, the Abaqus simulation is used. By optimizing the parameters of the radius of the cone top arc, we analyzed the changing law of the parameters of large-diameter D30 series conical PDC teeth, such as the equivalent force, impact force, and energy absorption of the conical teeth during the impact process, and optimized the best structure of the conical PDC teeth. After being subjected to a high temperature and high pressure, we synthesized the specimen for impact testing and analyzed the PDC layer crack extension and fracture failure. The findings reveal the emergence of a stress ring below the compacted area of the conical tooth. As the radius of the cone top arc increases, so does the area of the stress ring. When R ≥ 10 mm, the maximum stress change is minimal, and at R = 10 mm, the stress change in its top unit is relatively smooth. Optimal impact resistance is achieved, withstanding a total impact work value of 7500 J. Extrusion cracks appear in the combined layer part of PDC layers I and II, but the crack source is easy to produce in the combined layer of PDC layer II and the alloy matrix and extends to both sides, and the right side extends to the surface of the conical tooth in a “dragon-claw”. The failure morphology of the conical teeth includes ring shedding at the top of the PDC layer, the lateral spalling of the PDC layer, and the overall cracking of the conical teeth. Through this study, we aim to promote the popularization and application of large-diameter conical PDC teeth in the field of engineering rotary excavation.

## 1. Introduction

Since the early 1970s, when Polycrystal Diamond Compact (PDC) was first proposed by General Electric Company (GE) [1,2], domestic and foreign experts and scholars have been conducting in-depth explorations and research on the comprehensive performance of PDC teeth. By optimizing the tooth structure, material ratio, and forming process, its wear resistance and impact resistance are enhanced to improve its rock-breaking efficiency and extend the service life of the tool. Conical PDC teeth were developed by Hall and Durrand [1] in 2010, and compared with the traditional carbide teeth, the impact resistance was improved by 3~8 times and the wear resistance by 1~2 times [3], which greatly improves the breaking efficiency in highly abrasive hard rock and sandstone formations.

In recent years, in the research on the impact resistance of PDC teeth (composite pieces) in terms of their diamond grain size and content, FuXiao Zhang [4] conducted impact tests on five kinds of PDC composite pieces with different diamond grains, judged the performance according to the shipping time, and analyzed the fracture morphology of each composite piece by scanning electron microscopy, and the results showed that the finer the diamond grains were, in conjunction with the coarser the WC grains were, the better the impact resistance performance was. Valentine K. et al. [5] studied the fatigue behavior and failure of PDC composite pieces under continuous impact loading and observed the impact cracks inside the composite piece tool by scanning electron microscopy. They found that a given range of coarser-grained polycrystalline diamond layers has better impact resistance, and under continuous impact, cracks will be generated and extended intermittently until failure. Yuanyuan Li et al. [6] used the lattice point-spring model to analyze the impact test of diamond–silicon carbide superhard composites with different volume fraction ratios and concluded that the lowest damage degree was achieved when the volume content of diamond particles was 70%.

In their research on improving the comprehensive performance of conical PDC teeth, Jialiang Wang [7] and others used a falling hammer impact testing machine loaded with a high-circumference fatigue mode to consider the impact power, ball surface area, WC-Co cemented carbide teeth substrate thickness, and thickness of the PCD layer for the impact resistance of the teeth. The results show that the bigger the impact power, the higher the crushing rate; when the impact power is the same, the bigger the surface area of the ball, and the lower the crushing rate. When the impact power is the same, the larger the surface area of the ball, and the lower the crushing rate; the greater the thickness of the WC-Co tungsten carbide tooth substrate, and the smaller the thickness of the PCD layer, the better the impact resistance of the ball teeth. Yuanxiu Sun [8] optimizes and improves the parameter aspects affecting the performance of conical PDC teeth, from changes in the main parameters such as the diameter of the substrate, the cone top angle, the cone top radius, and so on, to an in-depth study of the impact on the impact resistance and wear resistance of the conical teeth. Chaowei He [9], in their study of the larger-diameter conical PDC teeth, through a finite element simulation combined with experiments, the larger-diameter conical PDC teeth for impact resistance analysis and cutting analysis, through the adjustment of their cone top angle and cone top radius structural parameters, preferred the better performance of the conical teeth.

However, in engineering rotary excavation, due to complex geological conditions, conical PDC teeth are often subjected to alternating loads and are prone to tooth tip wear, tooth body cracking, chipping, deformation [10,11,12,13], and other forms of failure, which reduces the impact resistance and service life of the conical PDC teeth and affects the productivity of the engineering rotary excavation [14], and the comprehensive performance of the conical PDC teeth still needs to be further improved. At present, regarding the design and analysis of conical PDC teeth, there are a large number of literature studies on the diameter of 14~16 mm conical teeth, ball teeth, and PDC pieces. Relying on the design criteria of cemented carbide teeth [15], due to the harsh molding conditions of large-diameter conical PDC teeth and the rotary excavation of highly abrasive hard rock, super-hard rock, and complex rock formations, large-diameter (D ≥ 20 mm) conical PDC teeth are needed to enhance their wear resistance and impact resistance, but there are fewer studies on the effects of the structural parameters of large-diameter conical PDC teeth on their impact resistance performance. Therefore, there is an urgent need to study the impact resistance of large-diameter conical PDC teeth. In this paper, finite element simulation is used to optimize the structural parameters of large-diameter D30 conical PDC teeth with different radii of the conical arc, and then high-temperature and high-pressure molding specimens are formed, and an impact resistance analysis is carried out on the specimens to further analyze the crack extension of the PDC layer and the overall distribution law [16]. This provides theoretical support for the design of large-diameter conical PDC teeth and promotes the popularization and application of large-diameter conical PDC teeth in the field of rotary excavation.

## 2. Simulation and Test Conditions

### 2.1. Conical PDC Tooth Structure Parameters

**Figure 1:**
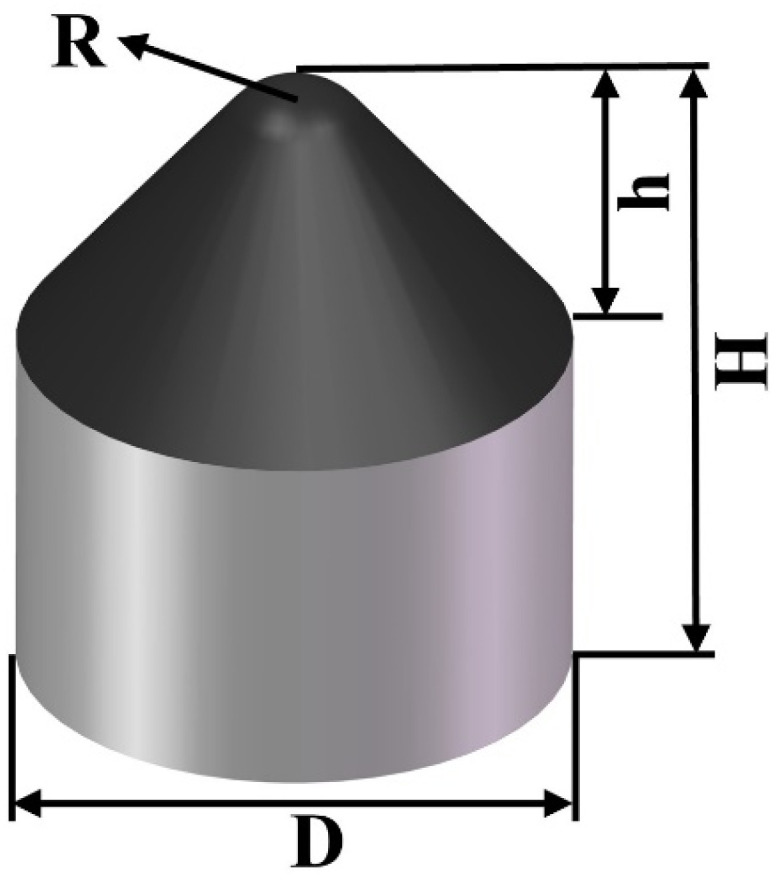
Geometrical parameters of conical PDC teeth.

**Figure 2:**
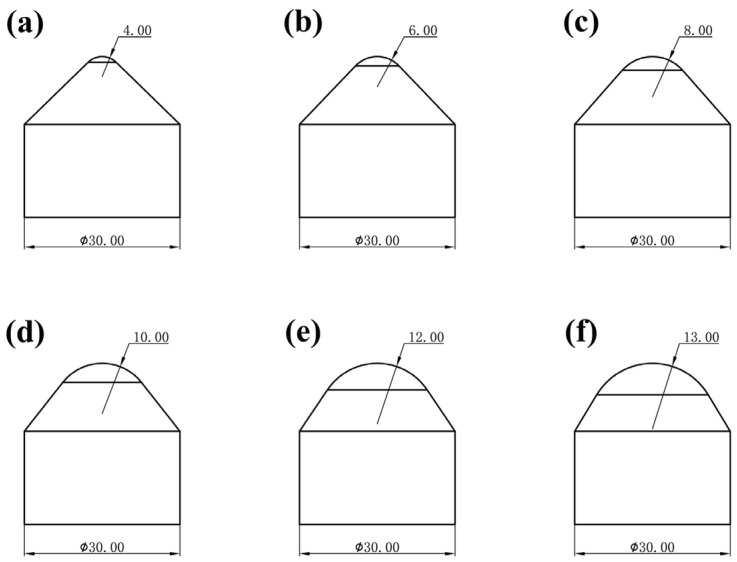
Structural parameters of D30 series conical PDC teeth ((**a**) R = 4 mm; (**b**) R = 6 mm; (**c**) R = 8 mm; (**d**) R = 10 mm; (**e**) R = 12 mm; (**f**) R = 13 mm).

The main structural parameters affecting the performance of conical PDC teeth include the radius of the top arc of the cone, R, the height of the top of the cone, h, the total height of the taper, H, and the diameter of the tungsten carbide substrate, D, as shown in Figure 1. In this test, a conical PDC tooth with the diameter of the tungsten carbide substrate, D, of 30 mm and the radius of the top arc of the cone of 4 mm, 6 mm, 8 mm, 10 mm, 12 mm, and 13 mm, was used with the ratio of the height of the top of the cone, h, and its overall height, H, of the cone remaining unchanged. The specific structure is as shown in Figure 2a–f. According to the working condition of the large-diameter conical PDC teeth, we set the impact parameters and analyzed the vertical impact on the conical PDC teeth.

### 2.2. Boundary Condition Loading for Simulation

The three-dimensional model was established by Solidworks, and the file was converted into .Stp format and imported into Abaqus 2020 software for impact simulation. The material selected for the conical PDC teeth is polycrystalline diamond, with a density of 3520 kg/m^3^, modulus of elasticity of 8.9 × 10^5^ MPa, Poisson’s ratio of 0.07, and a compressive strength of 7600 MPa [17,18]. The material selected for the impact block is 45# steel, with a density of 7850 kg/m^3^, modulus of elasticity of 2.06 × 10^5^ MPa, and Poisson’s ratio of 0.3 [19].

**Figure 3:**
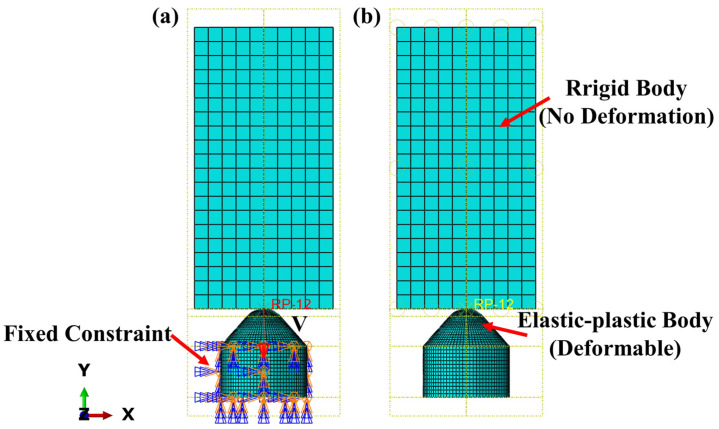
Schematic diagram of boundary condition setting and meshing ((**a**) load setup diagram; (**b**) interaction setup diagram).

The conical PDC teeth contact the impact block during assembly, and the impact starts when contact is made; the impact time is set to 3 × 10^−4^ s; the impact block is set to be a rigid body; the alloy matrix part is set to be at a fixed constraint; and the conical part of the conical PDC teeth is set to be an elastic–plastic body. To improve the accuracy of the model and efficient calculation, the conical PDC teeth are finely divided. The mesh size of the conical arc part of the conical PDC tooth is set to 0.5, the mesh size of the rest of the conical part is set to 0.8, the mesh size of the alloy matrix part is set to 1.3, the cell type is C3D8R, and the total number of cells is 23,244; the mesh size of the impactor block is set to 5, and the total number of cells is 2000, as shown in Figure 3.

### 2.3. Field Tests and Equipment

**Figure 4:**
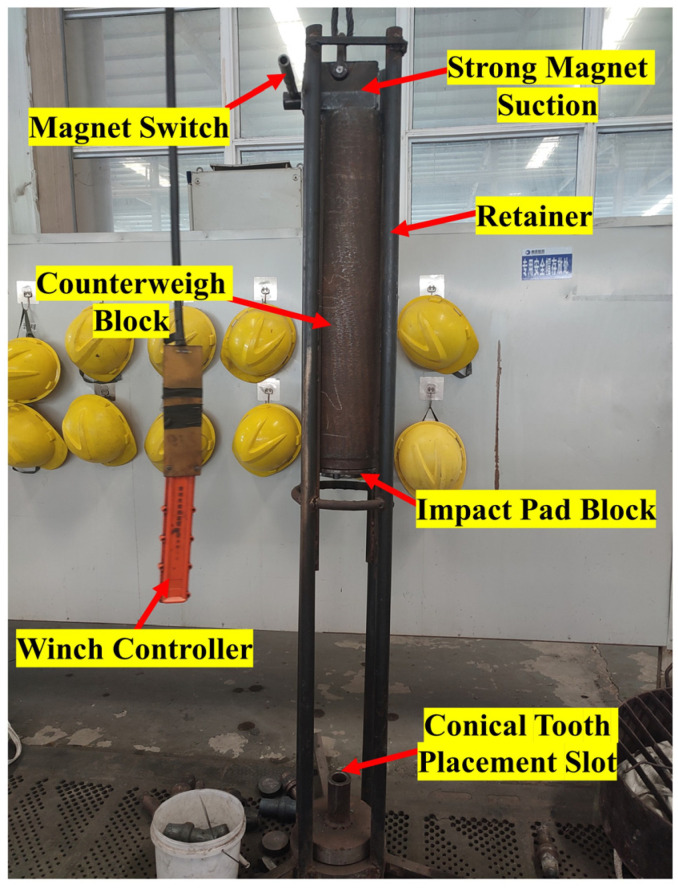
The overall structure of the impact tester.

The conical PDC teeth with superior impact resistance were simulated, and the PDC layer [20] with a layer thickness of 2~3 mm was synthesized on the cemented carbide substrate after being subjected to high temperature and high pressure. The PDC layer utilizes a dual-layer sample preparation technique, taking advantage of the gradient material, and the specimen was subjected to an impact test using the homemade impact testing machine. The overall structure of the testing machine is shown in Figure 4, and its main parts include a retainer, impact pad block, counterweight block, strong magnetic suction, winch controller, and conical tooth placement slot. The conical PDC teeth are mounted in the corresponding truncated tooth holders and placed in the truncated tooth placement slot for vertical impact. The mass of the impact block is 150 kg, the height of the impact is 0.5 m, the impact block adopts free-fall motion, the speed of the impact block in contact with the conical PDC teeth is calculated to be 3.1305 m/s, and the single impact work is set to 750 J.

**Figure 5:**
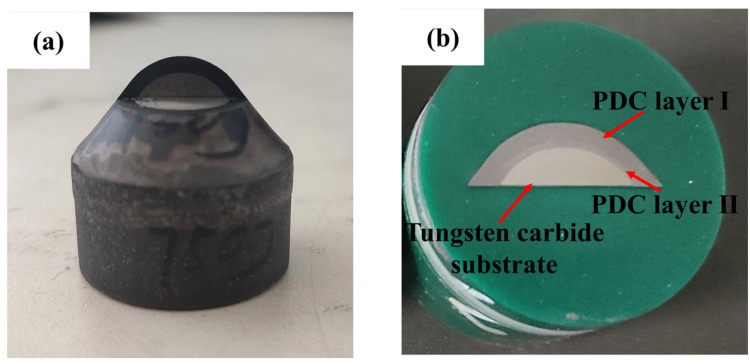
Specimen after wire cutting ((**a**) wire cutting method; (**b**) insertion of samples).

Subsequently, the conical PDC tooth specimen after the impact test was cut and made into samples: the HSQ2C EDM wire cutting machine was used to wire cut the impact area of the conical tooth as shown in Figure 5a, the ZXQ-5H automatic inlay machine was used to inlay the sample after wire cutting, and the MoPao3S automatic lapping/polishing machine was used to grind and polish the samples as shown in Figure 5b, and the sample was then polished by using an OYA-1048 ultrasonic cleaner with acetone to remove impurities on the specimen surface and observe the crack extension and the overall cracking of the PDC layer. Acetone was added through an OYA-1048 ultrasonic cleaner to remove the impurities on the surface of the specimen, and the specimen was examined using a JSM-6380LA scanning electron microscope (SEM) to observe the crack extension and overall distribution of the PDC layer. The parameters of the equipment used above are shown in Table 1.

## 3. Simulation Results and Analysis

### 3.1. Equivalent Stress Maps and Top Cell Stress Analysis

**Figure 6:**
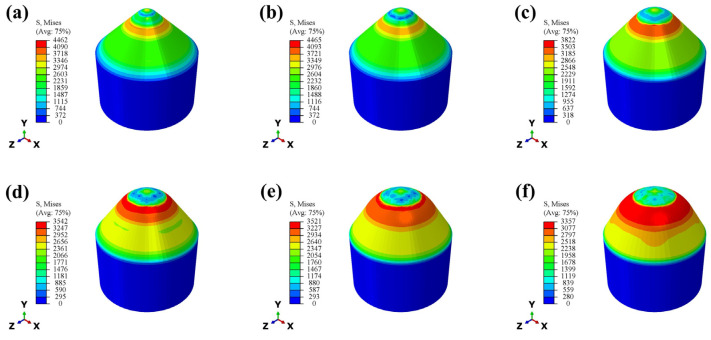
Mises cloud of equivalent stresses for each conical tooth ((**a**) R = 4 mm; (**b**) R = 6 mm; (**c**) R = 8 mm; (**d**) R = 10 mm; (**e**) R = 12 mm; (**f**) R = 13 mm).

By optimizing the parameter value of the radius of the top arc of the cone (R) and analyzing the effect of the different radii of the top arc of the cone on the impact resistance of the conical PDC teeth, the equivalent stress Mises cloud diagrams of each conical tooth are extracted, as shown in Figure 6. The appearance of a stress ring at the lower position of the compacted area [21] of the conical PDC teeth during impact is evident, which is due to the generation of the impact stress wave [22,23] accompanying the impact at the time of the impact. The impact force is transmitted from the compacted region to the surrounding area and is transmitted relatively uniformly through the conical tooth shape. Figure 6 shows that the maximum stress ring of the conical teeth appears in roughly the same location, but the size (area) is different. With the increase in the radius of the top arc of the cone, the maximum value of the stress is reduced, but the stress ring area increases. This is because the impact load in the impact process is vertically downward and due to the circumferential decomposition of the ring. The greater the radius of the top arc of the cone of the PDC tooth volume and the greater the area of contact, the more the stress ring of the unit area of the impact load is reduced. The impact load per unit area of the stress ring decreases because the impact load is decomposed in the circumferential direction at a rate greater than that in the axial direction [24], increasing the area of the stress distribution region decomposed in all directions.

**Figure 7:**
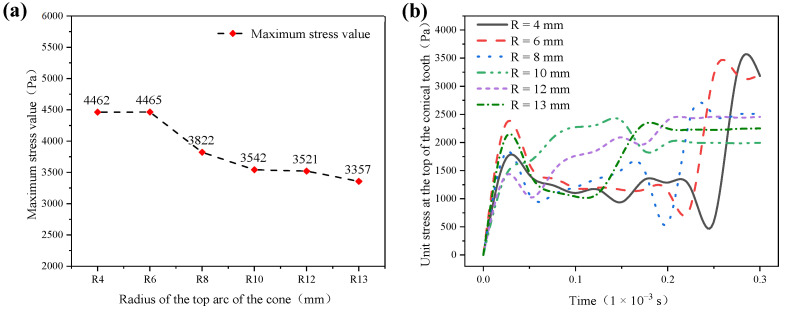
Maximum stress value of each conical tooth and change in stress in the top unit ((**a**) maximum stress value of each conical tooth; (**b**) stress change curve of the top unit of each conical tooth).

Through extracting the maximum value of stress in the stress ring, obtained as shown in Figure 7a, the overall trend decreases with the increase of the radius of the top arc of the cone. The inflection point begins to appear in the structure with R = 8 mm. With a larger decrease, the maximum stress in the structures with R = 10 mm, 12 mm, and 13 mm is relatively small, and the change is smooth. The maximum stress values of the conical PDC teeth with the radius of the top arc of the cone R = 4 mm, 6 mm, 8 mm, 10 mm, 12 mm, and 13 mm are 4462 Pa, 4465 Pa, 3822 Pa, 3542 Pa, 3521 Pa, and 3357 Pa, respectively. After extracting the stress change in the top unit of the conical teeth (the part in contact with the impactor block) and obtaining the stress change curve shown in Figure 7b, it can be seen that the R = 10 mm, 12 mm, and 13 mm structures have relatively large and smooth changes in the maximum stress. The stress change curve is shown in Figure 7b, which shows that the stress change trend of the top unit of the bevel tooth of the R = 10 mm structure is relatively smooth and the stress peak value is low.

### 3.2. Impact Force Analysis

**Figure 8:**
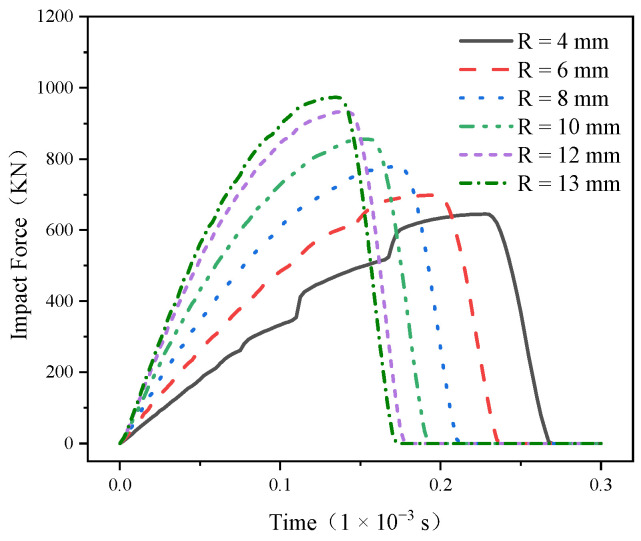
Impact force–time curve.

The main reason for the failure of conical teeth is the large impact force caused by alternating loads, which leads to the cracking, chipping, and crushing of conical teeth [25], so the analysis of the impact force is particularly important. The impact force–time curve of the truncated tooth is shown in Figure 8, and it can be concluded that the conical PDC tooth is divided into two phases during the impact process: the elastic deformation phase and the fracture phase, and its impact process is the impact contact between the rigid–elastic–plastic body [26]. Its elastic deformation stage is a rising quarter sine wave, but the fracture stage is an extremely steep downward straight line, and the impact force–time curve is smooth and without volatility during the whole impact process. This is because the PDC layer is a brittle material, and when the impact force exceeds the maximum limit value it can withstand, brittle fractures occur instantaneously. It can also be seen from Figure 8 that the vertical impact process impact force first increased to the peak and then quickly reduced to 0, and with the increase in the radius of the cone top arc, the peak impact force borne by the conical PDC teeth increased; the maximum (R = 13 mm) reached 973 KN. The R = 4 mm structure in Figure 8 reveals that a small portion of the peak impact force and a very small portion of the gradual rise can be attributed to the plastic deformation occurring within the alloy matrix section of the plastic deformer.

### 3.3. Analysis of Energy Absorption in Bevel Gears

Energy absorption is not only an important index to evaluate the energy absorption of the structure but also reflects the ability of the specimen to withstand the load. The impact work absorbed by the conical PDC teeth during the impact of this test is mainly composed of the kinetic energy of the impact block and the gravitational potential energy [9], according to which the impact work absorbed by the conical teeth can be calculated as follows:

(1) $$E_{X}=E_{CMAX}+mgS-E_{C},$$

where *E_X_* is the energy absorbed by the conical PDC teeth, J; *E_CMAX_* is the initial kinetic energy of the impact block, i.e., the initial impact work set for the test, J; *m* is the mass of the impact block, kg; *g* is the acceleration of gravity, m/s^2^; *S* is the displacement distance of the impact block, m; *E_C_* is the kinetic energy of the impact block, J.

**Figure 9:**
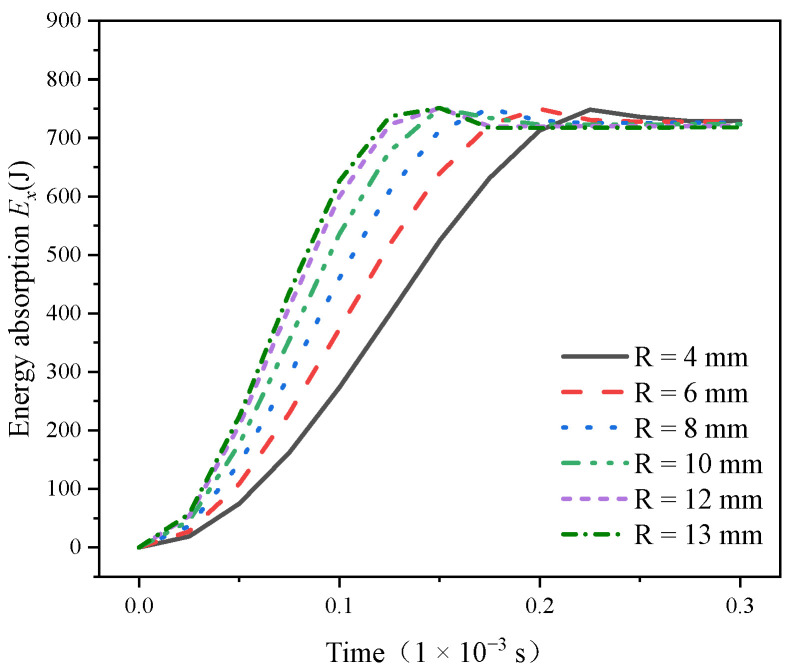
Energy absorption *E_x_*–time curve.

Inserting the above data into Formula (1) enabled us to calculate the conical PDC teeth energy absorption time curve, shown in Figure 9. It can be seen that the D30 conical PDC teeth’s energy absorption curve trend is the same; with the increase in the impact contact time, the cone tooth’s absorbed energy from the initial value gradually increased to the peak value, followed by a slight decline, and eventually stabilized. The extracted conical teeth energy absorption data are shown in Table 2; the results show that the peak value of the energy absorbed by the conical PDC teeth increases with the increase of the radius of the top arc of the cone, the maximum value (R = 13 mm) is 751.2088 J, the peak time of appearance decreases with the increase of the radius of the top arc of the cone, and the peak time of appearance stabilizes at 0.15 × 10^−3^ s when R = 10~13 mm. Combined with Figure 9, it can be seen that the energy absorption efficiency of the conical PDC teeth increases with the increase of the radius of the top arc of the cone. This shows that under the same impact work, the larger the radius of the top arc of the cone, the larger the peak value of the energy absorbed by the conical PDC teeth, the faster the energy absorption efficiency, and the better the vertical impact resistance, which shows that the energy absorbed by the conical PDC teeth is related to the volume and mass of the conical teeth, and under the condition of the constant ratio of the cone height and the total height, with the increase in the mass and volume of the conical PDC teeth, in the process of the impact, the ability to absorb the impact work is enhanced, and the overall fluctuation is lower when the R ≥ 10 mm after the overall fluctuation is small; its law coincides with the change rule of the impact force-time in Section 3.2. The decrease in the energy absorption of the conical teeth at the tail end of the energy absorption–time curve in Figure 9 is due to the deformation of the conical PDC teeth that dissipates a small portion of the kinetic energy of the impact block at the end of the time period analyzed in the impact simulation.

## 4. Test Results and Analysis

### 4.1. Impact Resistance of Conical PDC Teeth

**Figure 10:**
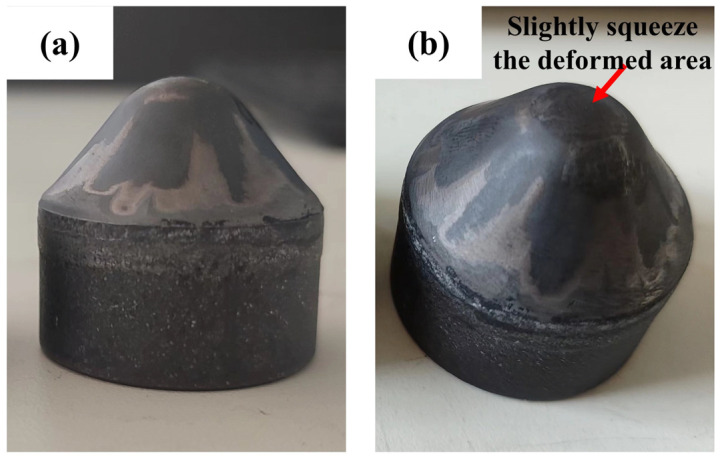
Synthetic specimen at high temperature and high pressure ((**a**) before impact; (**b**) after impact).

According to the simulation results and combined with the working conditions of conical PDC teeth in rotary excavation projects, from the comprehensive consideration of improving the efficiency of rotary excavation and extending the teeth’s service life, the comprehensive impact resistance performance of the R = 10 mm structural conical PDC teeth is excellent. High-temperature and high-pressure synthesized specimens were made [27]. As shown in Figure 10a, the surface of the conical teeth is smooth and neat, and the synthesis process did not appear to cause tooth cracks, chipping, delamination, or other defects [28,29]. Subsequently, the vertical impact test was carried out. After 10 tests, the total impact work of the specimen was 7500 J (single impact work is 750 J); after the impact, it was not broken, but its head has a slight extrusion deformation as shown in Figure 10b. The predicted number of impact times is consistent with the expected requirements, indicating that the use of a reasonable structure can be prepared, with the excellent impact resistance of the large-diameter conical PDC teeth, to meet the requirements of application with highly abrasive hard rock, super-hard rock, and complex rock formations. The application of PDC teeth in highly abrasive hard rock, ultra-hard rock, and complex rock formations is possible.

### 4.2. Generation of Cracks in the PDC Layer

**Figure 11:**
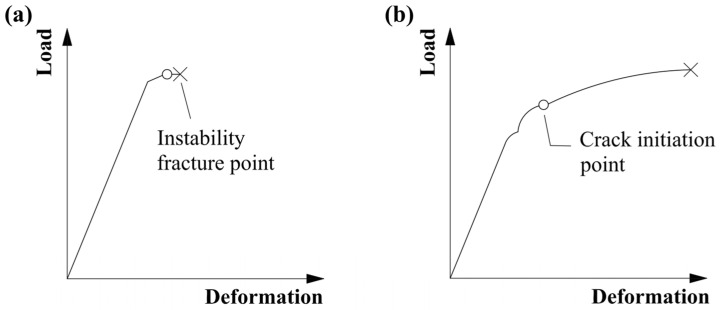
Load–deformation relationship for material fracture ((**a**) brittle fracture; (**b**) ductile fracture).

In the process of rotary excavation, conical tooth cracking, chipping, and crushing are the main forms of conical PDC tooth failure; the cause of workpiece cracking is largely determined by its material properties and is closely related to the microstructure of the material. Due to the microstructure of polycrystalline diamond being face-centered cubic stacking, the structure is stable, and the PDC layer has a high level of hardness and brittleness, which is prone to brittle fracture under a large impact load [30,31,32]. Figure 11 shows the load–deformation relationship graphs for different fracture modes; brittle fractures occurs when the load reaches the maximum value that the material can withstand to reach the destabilizing fracture point, followed by the instantaneous fracture and failure of the workpiece. The toughness of the fracture increases when the load reaches the crack initiation point state. As the load continues to increase, the crack is then extended until the fracture occurs, resulting in workpiece failure. The conical PDC tooth displays brittle fracture, and the whole impact process is divided into two stages: the elastic deformation stage and the fracture stage, which coincide with the law of the impact force–time curve simulated by finite element simulation in Section 3.2.

### 4.3. Crack Expansion of the PDC Layer

**Figure 12:**
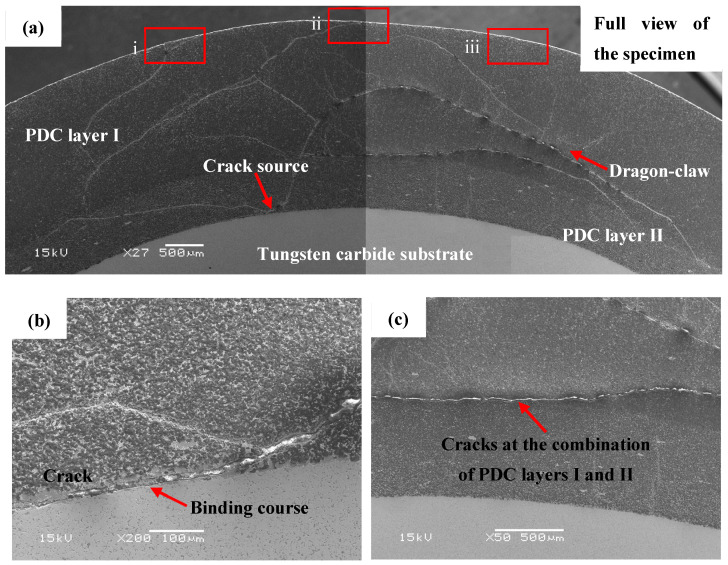

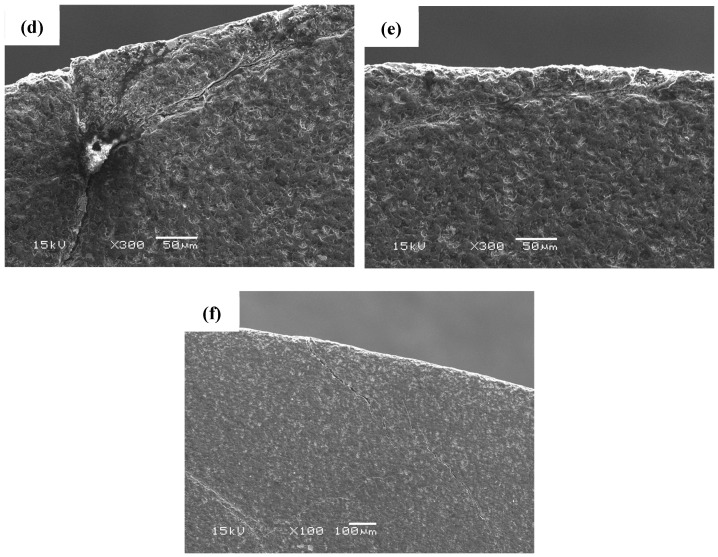
Scanning electron microscope image of the specimen (SEM) ((**a**) full view of the crack; (**b**) magnified view of the crack source; (**c**) crack at the interface of PDC layers I and II; (**d**) magnified view at i; (**e**) magnified view at ii; (**f**) magnified view at iii).

Figure 10: The conical PDC teeth were not broken, but cracks were produced inside the PDC layer due to multiple impacts. As shown in Figure 12a for the specimen scanning electron microscopy, the distribution of cracks in the PCD layer I, PCD layer II, and the distribution of the alloy matrix can be seen. The overall distribution of cracks presents a “dragon-claw” on the left and right, as shown in Figure 12a. Figure 12a also shows that the crack distribution density of PDC layer I is larger, which is because PDC layer I contains more brittle material, diamond, leading to the rapid expansion of cracks [33,34,35]. The conical tooth head compaction region is initially subjected to a higher impact force. As the number of impacts increases, the impact force follows a designed arc downward and around the conduction path. In this process, stress concentration occurs in both PCD layer II and the bonding layer between alloy matrix and polycrystalline diamond due to differences in physical properties resulting from high temperature and high pressure synthesis of base alloy materials and polycrystalline diamond properties. These residual stresses lead to extrusion cracks in PCD layer II and the bonding layer [36], as shown clearly in Figure 12b when magnifying crack sources observed in Figure 12a.

With the increase in the number of impacts, the cracks were extended from the crack source to the left and right diagonally above, and the cracks distributed to the left stopped at the interface of PDC layer I and PDC layer II. The cracks extending from the crack source to the right diagonally above led to several upward river-like small cracks, as shown in Figure 12c, and due to the load conduction during the impacts, the cracks were extended toward the region of higher stress, and the cracks were then extended to the head of the conical gears. An shown in Figure 12d–f, an enlarged view of the crack, the surface cracks generated in the region coincide with the location of the maximum stress ring in the previous Section 3.1; the specimen withstood 10 impacts without rupture, however, upon further increasing the impact force, laminar detachment occurred in the area, leading to failure of the conical tooth.

### 4.4. Impact Failure Morphology

**Figure 13:**
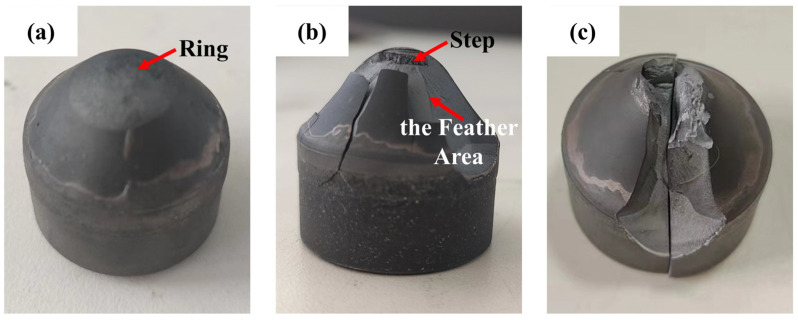
Failure of a conical PDC tooth by impact crushing ((**a**) failure of the top ring of the PDC layer; (**b**) failure of the side spalling of the PDC layer; (**c**) failure of the overall cracking of the conical tooth specimen).

The crushing process of the conical PDC teeth includes crack initiation, crack extension, and final crushing and shedding. The crushing failure morphology of the conical PDC tooth specimens that are continuously impacted until failure occurs was analyzed. The morphology of the conical teeth after impact failure is shown in Figure 13, and there are three main forms, which are the annular shedding failure on the top of the PDC layer, the lateral spalling failure of the PDC layer, and the overall cracking failure of the conical teeth. The PDC layer is a brittle material, and when brittle crushing occurs according to Griffith’s theoretical equations [37,38,39], it can be seen that the fracture stress increases with the increase in the surface energy consumed by the crack expansion.

(2) $$\sigma_{c}=\sqrt{\frac{2\gamma E}{\pi a\left(1-v^{2}\right)}}$$

where σ_c_ represents the fracture stress, MPa; γ represents the surface energy consumed per unit area of crack extension, J; *E* represents the Young’s modulus; *a* is the crack radius, m; *v* is Poisson’s ratio.

When the top ring-off failure of the PDC layer occurs, as depicted in Figure 13a, it is primarily attributed to the elevated stress levels experienced by the maximum stress ring beneath the compaction area, rendering it susceptible to rupture. The subsequent multiple impacts result in crack propagation over time, ultimately leading to complete dislodgement and failure of the uppermost portion of the PDC layer. When the failure form for the PDC layer is a lateral spalling failure, this situation is more serious. As can be seen from Figure 13b, the PDC layer occurs in a large area of broken spalling, and the deconstruction crack expansion process is accompanied by a step. Due to the small head of the conical tooth compaction region at the location of stress, the maximum stress ring below it ruptures, and with the increase in the number of impacts, the cracks propagated through the radial feather region in the direction of tooth shape [40]. With the increase in the number of impacts, the cracks expand along the radial feather zone in the direction of the tooth shape, and the cracks produced by the dissociated surfaces in the dynamic impact process are branched and combined. The impact force is transformed into the kinetic energy of crack expansion, which finally leads to the spalling of the PDC layer on the surface of the conical tooth. When the overall cracking failure of the conical tooth, as shown in Figure 13c, occurs, this situation is even more serious because the impact force is too large between the PDC layer and the matrix part of the extrusion cracking, many times the dynamic impact, so that the PDC layer is subjected to serious damage, and the compressive stress caused by the PDC layer II and the alloy matrix bonding layer of the seam cracks between the extension is extended, ultimately leading to the complete cracking failure of the whole matrix.

## 5. Conclusions

(1) In the large-diameter D30 series conical PDC teeth in the vertical impact process, the maximum stress ring appears below the compaction area of the conical teeth, and the maximum stress ring area increases with the increase in the radius of the top arc of the cone.
(2) The large-diameter D30 series conical PDC teeth with an increase in the radius of the top arc of the cone can withstand the maximum impact force, peak energy absorption, and energy absorption efficiency, and the R ≥ 10 mm structure of the maximum stress and peak energy absorption of the conical teeth tends to stabilize.
(3) The R ≥ 10 mm structural bevel teeth of the maximum stress value are relatively small, and the stress value of the R = 10 mm structural bevel teeth of the top unit changes more smoothly. The high-temperature and high-pressure synthetic specimens with impact resistance are excellent, and a total impact work of 7500 J did not occur.
(4) When the conical PDC teeth were subjected to continuous impact, extrusion cracks were caused in the combined layer of PDC layer II and the alloy matrix and expanded to both sides, with the left side expanding to the combined layer of PDC layer I and PDC layer II and the right side expanding to the surface of the conical teeth in the form of a “dragon-claw”.
(5) The conical PDC teeth were continuously impacted until failure, and the failure morphology can be divided into ring peeling off the top of the PDC layer, flaking off the side of the PDC layer, and the overall cracking of the conical teeth.

## Figures and Tables

**Table 1:** Summary of equipment in use.

Equipment Name	Model Number	Manufacturer’s Name	Procurement Country
EDM Wire-cutting Machine	HSQ2C	Suzhou Sanguang Science Technology Co., Ltd.	Suzhou, China
Automatic Inlay Machine	ZXQ-5H	Laizhou Huayin Test Instrument Co., Ltd.	Laizhou, China
Automatic Grinding/Polishing Machine	MoPao3S	Laizhou Weiyi Experimental Machine Manufacturing Co., Ltd.	Laizhou, China
Ultrasonic Cleaner	OYA-1048	Suzhou OYA Ultrasonic Equipment Co., Ltd.	Suzhou, China
Scanning Electron Microscope (SEM)	JSM-6380LA	Japan Electron Optics Laboratory Co., Ltd.	Japan

**Table 2:** Analysis of the absorbed energy of conical PDC teeth.

Impact Power/J	Radius of the Top Arc of the Cone/mm	Peak Energy Absorption/J	Peak Occurrence Time/(1 × 10^−3^ s)
750	R = 4	748.4091	0.225
	R = 6	749.3675	0.200
	R = 8	750.1123	0.175
	R = 10	750.6345	0.150
	R = 12	750.9841	0.150
	R = 13	751.2088	0.150

## Data Availability

The data presented in this study are available on request from the corresponding author. The data are not publicly available due to the structural parameters of the conical PDC teeth in this paper are confidential to the corresponding author and the associated organization.
